# PyPeakRankR: Reproducible Peak-Level Feature Extraction for Regulatory Element Ranking

**Published:** 2026-06-16

**Authors:** Saroja Somasundaram, Nelson J. Johansen, Trygve E. Bakken, Jeremy A. Miller

**Affiliations:** 1 Allen Institute for Brain Science, Seattle, WA, USA

**Keywords:** ATAC-seq, genomics, regulatory elements, bioinformatics, peak ranking, enhancer, Python

## Abstract

High-throughput chromatin accessibility assays such as ATAC-seq generate thousands of candidate regulatory elements (peaks), yet no standardized tool exists for assembling the diverse quantitative features needed to prioritize peaks for functional validation. Here we present PyPeakRankR, an open-source Python package that extracts peak-level features, namely BigWig signal summaries, GC content, PhyloP conservation scores, distribution moments (kurtosis, skewness, bimodality), and cell-type specificity rankings, into a single reproducible peak by feature matrix stored as a tab-separated values (TSV) file. PyPeakRankR separates deterministic feature extraction from downstream ranking, enabling transparent benchmarking of prioritization strategies on the same upstream data. The package provides both a command-line interface and a matching Python API, supports cross-assembly scoring via liftOver, and runs in minutes on thousands of peaks. PyPeakRankR was validated in the Brain Initiative Cell Census Network (BICCN) community challenge, where its predecessor PeakRankR ranked among the top 3 of 16 methods for cell-type-specific enhancer prediction. In a recent basal ganglia study, PyPeakRankR was used within the Cross-species Enhancer Ranking Pipeline (CERP) to identify enhancer-AAV tools achieving greater than 70% on-target specificity across cell types. PyPeakRankR is freely available under the MIT license at https://github.com/AllenInstitute/PeakRankR/tree/python-package.

## 1 Introduction

High-throughput chromatin accessibility assays such as ATAC-seq (Buenrostro et al. 2013) generate large sets of candidate regulatory elements called “peaks.” Downstream analyses require prioritizing peaks across cell types, experimental conditions, or species to identify the most biologically relevant candidates for functional validation. However, peak prioritization workflows are frequently implemented using ad hoc scripts with inconsistent feature definitions and aggregation strategies, limiting reproducibility and cross-study comparability.

Prioritizing genomic peaks across cell types requires combining multiple features: signal intensity, sequence properties such as GC content, evolutionary conservation (PhyloP (Pollard et al. 2010)), and higher-order signal statistics. These features are typically computed using custom per-project scripts that vary across laboratories, complicating benchmarking and cross-study integration. This gap particularly affects computational biologists working with single-cell ATAC-seq (scATAC-seq) or bulk ATAC-seq data who need to systematically prioritize peaks for experimental follow-up, especially for enhancer discovery or adeno-associated virus (AAV) tool design.

Existing genomics tools each address part of the problem but none assembles a unified, portable feature matrix. Peak callers such as MACS2 (Zhang et al. 2008) identify open chromatin regions but rank peaks only by fold change or p-value, reflecting signal strength rather than cell-type specificity. A peak with high fold change may be active across many cell types and therefore a poor candidate for cell-type targeted AAV tools. Differential accessibility tools such as ArchR (Granja et al. 2021) test for cell-type enrichment but operate within their own data model and do not produce portable, tool-agnostic feature tables. Annotation tools such as GREAT (McLean et al. 2010) link peaks to genes but do not score chromatin features. At the library level, pyBigWig (Ramírez and Diehl 2020) provides low-level BigWig access without peak-level aggregation, deepTools (Ramírez et al. 2016) computes matrix summaries oriented toward visualization, and pyfaidx (Shirley et al. 2015) enables FASTA access without a genomics feature pipeline.

Table 1 summarises feature coverage across these tools.

PyPeakRankR fills this gap by combining pyBigWig, pyfaidx, and SciPy (Virtanen et al. 2020) into a flexible CLI pipeline that assembles heterogeneous features into a single reproducible TSV table.

## 2 Software design

PyPeakRankR is built around three core design decisions:

### Table-first, flexible pipeline.

Every subcommand reads an existing tab-separated values (TSV) file and appends columns without modifying the peak coordinates. Users can run any subset of steps or add custom columns; the table remains valid throughout. The table-first design enables incremental extension.

### Separation of feature extraction from ranking.

Feature extraction is deterministic: given the same inputs and the same BigWig files, the same table is always produced. Ranking is deliberately left to the user or to the rank-specificity subcommand, which implements one well-defined ranking formula but is not the only option. This separation means benchmarking studies can compare ranking strategies using the same upstream feature matrix, which is exactly how PyPeakRankR was used in the Brain Initiative Cell Census Network (BICCN) challenge (Johansen et al. 2025).

### Command-line interface (CLI) + Python API parity.

Every subcommand wraps a public Python function (init_table, add_signal, add_gc, add_phylop, add_moments, rank_by_specificity), so the tool works equally in shell pipelines and Python notebooks without reimplementing logic.

The specificity ranking formula computes the ratio of target group signal to mean background signal, then min-max normalises to [0,1]. This matches the CERP pipeline (Wirthlin et al. 2026) and the ATAC-specificity metric validated in the BICCN challenge (Johansen et al. 2025).

Each feature has a distinct biological rationale. GC content is lower in active enhancers than in promoters or bulk genomic DNA, reflecting differences in nucleosome occupancy. PhyloP conservation (Pollard et al. 2010) identifies peaks under cross-species purifying selection. Signal distribution moments, including kurtosis (sharpness), skewness (asymmetry), and bimodality (Sarle’s coefficient), are motivated by Lu et al. (Lu et al. 2015), who showed these shape features distinguish enhancers from promoters in ChIP-seq data more reliably than signal intensity alone. The table-first design is directly extensible: future columns could include sequence model importance scores (e.g., from Borzoi or Enformer) or spatially resolved scores from multiplexed error-robust fluorescence in situ hybridization (MERFISH), integrating epigenomic and spatial context in one reproducible matrix.

## 3 Implementation

PyPeakRankR is implemented in Python (≥3.9) with the following dependencies: pandas (pandas development team 2020) for tabular data handling, NumPy (Harris et al. 2020) for numerical computation, pyBigWig (Ramírez and Diehl 2020) for BigWig signal extraction, pyfaidx (Shirley et al. 2015) for FASTA sequence access, and SciPy (Virtanen et al. 2020) for statistical distribution metrics. The package is installable via pip from GitHub and includes a pypeakranker CLI, unit tests, and example data. Source code is available at https://github.com/AllenInstitute/PeakRankR/tree/python-package under the MIT license.

## 4 Results

### 4.1 BICCN community challenge validation

PyPeakRankR extends the R package PeakRankR, which used a minimal set of three features (ATAC specificity, signal magnitude, and peak coverage) in the BICCN Community Challenge (Johansen et al. 2025). PeakRankR ranked among the top 3 of 16 methods for predicting cell-type-specific enhancers in the mammalian cortex. PyPeakRankR re-implements and expands this approach in Python to integrate directly with sequence models and modern genomics workflows.

### 4.2 Cross-species enhancer-AAV toolkit

In a recent basal ganglia study from our group (Wirthlin et al. 2026), PyPeakRankR was used within the Cross-species Enhancer Ranking Pipeline (CERP) across multiple basal ganglia (BG) cell types in mouse and macaque. The composite feature rankings outperformed conventional fold-change approaches, and the resulting enhancer-AAV tools achieved *>*70% on-target specificity across cell types, with exemplary enhancers exceeding 90%.

These results establish direct experimental utility of the feature extraction and ranking approach implemented in PyPeakRankR.

## 5 Discussion

PyPeakRankR addresses the lack of a standardized, portable tool for peak-level feature extraction in regulatory genomics. By separating deterministic feature computation from ranking logic, PyPeakRankR enables reproducible benchmarking of prioritization strategies. Its validation in the BICCN community challenge and application in cross-species enhancer-AAV design demonstrate practical utility for enhancer discovery workflows.

The table-first design is directly extensible: future columns could include sequence model importance scores (e.g., from Borzoi or Enformer) or spatially resolved scores from MERFISH, integrating epigenomic and spatial context in one reproducible matrix.

## Figures and Tables

**Figure 1:**
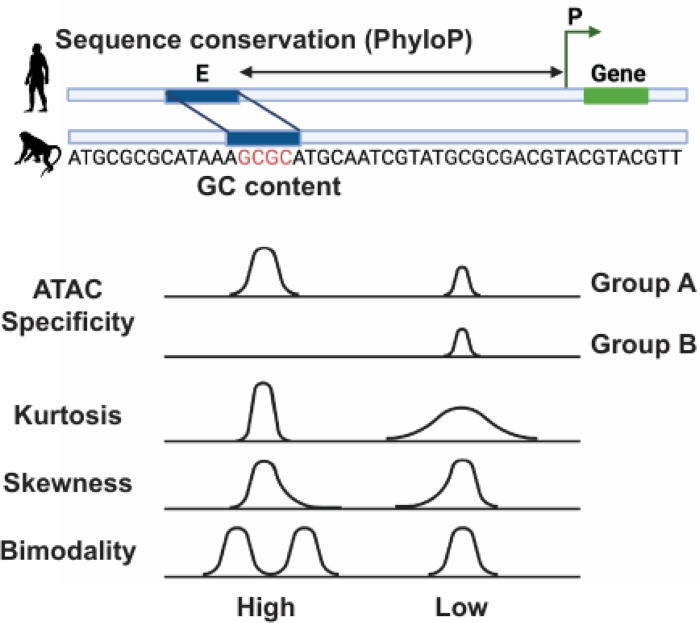
Features collected by PyPeakRankR for each candidate peak: GC content, PhyloP conservation, ATAC specificity, and signal distribution moments (kurtosis, skewness, bimodality). Figure adapted from Wirthlin et al. (2026).

**Table 1:** Feature coverage across genomics tools. **✓**= supported natively; partial = limited or indirect support; – = not supported. ArchR portable output is partial because outputs are tied to the ArchR project object.

Tool	Peak signal	Cell-type specificity	GC content	PhyloP cons.	Signal moments	Portable TSV	CLI + Python API	Cross-assembly
PyPeakRankR	**✓**	**✓**	**✓**	**✓**	**✓**	**✓**	**✓**	**✓**
ArchR	**✓**	**✓**	–	–	–	partial	–	–
MACS2	**✓**	–	–	–	–	**✓**	**✓**	–
deepTools	**✓**	partial	–	–	–	**✓**	**✓**	–
GREAT	–	–	–	–	–	**✓**	web only	–
pyBigWig	low-level	–	–	–	–	–	**✓**	–
pyfaidx	–	–	via FASTA	–	–	–	**✓**	–

## Data Availability

PyPeakRankR is open source (MIT license) and available at https://github.com/AllenInstitute/PeakRankR/tree/python-package. The Zenodo archive is available at https://doi.org/10.5281/zenodo.15238527.

## References

[R1] Buenrostro J. D., Giresi P. G., Zaba L. C., Chang H. Y., & Greenleaf W. J. (2013). Transposition of native chromatin for fast and sensitive epigenomic profiling of open chromatin, DNA-binding proteins and nucleosome position. Nature Methods, 10(12), 1213–1218. 10.1038/nmeth.2688 24097267 PMC3959825

[R2] Granja J. M., Corces M. R., Pierce S. E., (2021). ArchR is a scalable software package for integrative single-cell chromatin accessibility analysis. Nature Genetics, 53(3), 403–411. 10.1038/s41588-021-00790-6 33633365 PMC8012210

[R3] Harris C. R., Millman K. J., van der Walt S. J., (2020). Array programming with NumPy. Nature, 585(7825), 357–367. 10.1038/s41586-020-2649-2 32939066 PMC7759461

[R4] Johansen N. J., Kempynck N., Zemke N. R., Somasundaram S., De Winter S., (2025). Evaluating methods for the prediction of cell-type-specific enhancers in the mammalian cortex. Cell Genomics, 5(6), 100879. 10.1016/j.xgen.2025.100879 PMC12230242 40403730

[R5] Lu Y., Qu W., Shan G., & Zhang C. (2015). DELTA: A distal enhancer locating tool based on AdaBoost algorithm and shape features of chromatin modifications. PLoS ONE, 10(6), e0130622. 10.1371/journal.pone.0130622 PMC4474808 26091399

[R6] McLean C. Y., Bristor D., Hiller M., (2010). GREAT improves functional interpretation of cis-regulatory regions. Nature Biotechnology, 28(5), 495–501. 10.1038/nbt.1630 PMC4840234 20436461

[R7] Pollard K. S., Hubisz M. J., Rosenbloom K. R., & Siepel A. (2010). Detection of nonneutral substitution rates on mammalian phylogenies. Genome Research, 20(1), 110–121. 10.1101/gr.097857.109 19858363 PMC2798823

[R8] Ramírez F., & Diehl S. (2020). pyBigWig: A Python extension for reading BigWig files. https://github.com/deeptools/pyBigWig

[R9] Ramírez F., Ryan D. P., Grüning B., (2016). deepTools2: A next generation web server for deep-sequencing data analysis. Nucleic Acids Research, 44(W1), W160–W165. 10.1093/nar/gkw257 27079975 PMC4987876

[R10] Shirley M. D., Ma Z., Pedersen B. S., & Wheelan S. J. (2015). Efficient “Pythonic” access to FASTA files using pyfaidx. 10.7287/peerj.preprints.970v1

[R11] pandas development team. (2020). pandas-dev/pandas: Pandas. 10.5281/zenodo.3509134

[R12] Virtanen P., Gommers R., Oliphant T. E., (2020). SciPy 1.0: Fundamental algorithms for scientific computing in Python. Nature Methods, 17(3), 261–272. 10.1038/s41592-019-0686-2 32015543 PMC7056644

[R13] Wirthlin M. E., Hunker A. C., Somasundaram S., (2026). A cross-species enhancer-AAV toolkit for cell type-specific targeting across the basal ganglia. bioRxiv, ahead of print. 10.64898/2026.02.23.706695

[R14] Zhang Y., Liu T., Meyer C. A., (2008). Model-based analysis of ChIP-seq (MACS). Genome Biology, 9(9), R137. 10.1186/gb-2008-9-9-r137 18798982 PMC2592715

